# Taxonomy and phylogeny of the genus *Gastrocentrum* Gorham (Coleoptera, Cleridae, Tillinae), with the description of five new species

**DOI:** 10.3897/zookeys.979.53765

**Published:** 2020-10-27

**Authors:** Ganyan Yang, Xingke Yang, Hongliang Shi

**Affiliations:** 1 College of Forestry, Beijing Forestry University, Beijing 100083, China Beijing Forestry University Beijing China; 2 Key Laboratory of Zoological Systematics and Evolution, Institute of Zoology, Chinese Academy of Sciences, Beijing, 100101, China Chinese Academy of Sciences Beijing China

**Keywords:** Australian region, female, morphology, Oriental region, systematics

## Abstract

The genus *Gastrocentrum* Gorham, 1876 is revised to include nine species. Five new species are described in this genus: *G. magnum***sp. nov.** (NE India), *G. regulare***sp. nov.** (Cameron Highlands, Malaysia), *G. xiaodongi***sp. nov.** (Gyirong, Xizang, China), *G. zayuense***sp. nov.** (Zayü, Xizang, China), and *G. gaoligongense***sp. nov.** (Fugong, Yunnan, China). *Gastrocentrum nitidum* Schenkling, 1916 is transferred to the genus *Tillus* as a new combination. All the species in this genus are described (except *G. brevicolle*), and a key is provided for their identification. Illustrations of male genitalia, female reproductive organs, and other important structures are provided. An interspecific phylogeny-estimate of *Gastrocentrum* is presented based on morphological data, with two main clades recognized: a clade containing *G. unicolor* and *G. laterimaculatum*, and a clade containing the remaining six species (the latter a polytomy consisting of *G. magnum***sp. nov.**, *G. dux*, and *G. regulare***sp. nov.**, and a well-supported sub-clade representing the remaining species). Additionally, the taxonomic and phylogenetic importance of female reproductive organs is discussed.

## Introduction

*Gastrocentrum* Gorham, a genus of checkered beetles distributed throughout the Oriental and Australian regions, was established by Gorham (1876) who erected it for *G. pauper* Gorham, from Luzon, Philippines. Later, Gahan (1910) transferred *Notoxus unicolor* White, 1849 (type locality: India), and *Tillus dux* Westwood, 1852 (type locality: Australia) to *Gastrocentrum*, synonymizing *G. pauper* with the former species. The description of three more species (viz., *G. nitidum* Schenkling, 1916 from Taiwan, *G. brevicolle* Pic, 1940 from Sri Lanka, and *G. laterimaculatum* Gerstmeier, 2005 from Malaysia) brought the total number of valid *Gastrocentrum* species to five.

Mawdsley (1999) briefly reviewed this genus, but apart from the type specimens, he had only examined specimens from India and Sri Lanka, and male genitalia were not compared. In our research, specimens from a wide range of Oriental and Australian regions were examined, and both male genitalia and female reproductive organs are dissected and compared. Thus, this paper aims to re-evaluate the species components of genus *Gastrocentrum*, describe five new species, and analyses infra-generic phylogenetic relationships; besides, discuss the significance of female reproductive organs in taxonomy.

## Materials and methods

### Taxonomic study

The materials used in this work are from the following collections:

**FBFU** College of Forestry, Beijing Forestry University, Beijing, China

**IZAS** Institute of Zoology, Chinese Academy of Sciences, Beijing, China

**MNHN** Muséum national d’Histoire naturelle, Paris, France

**NHMB** Naturhistorisches Museum, Basel, Switzerland

**NKME** Naturkundemuseum Erfurt, Germany

**NMPC** Národní Muzeum Přírodovědecké Muzeum, Prague, Czech Republic

**RGCM** Roland Gerstmeier Collection, Munich (deposited in Zoologische Staatssammlung München), Germany

**SDEI** Senckenberg Deutsches Entomologisches Institut, Müncheberg, Germany

**ZMAN** Zoological Museum Amsterdam, Naturalis Biodiversity Center, Leiden, Netherlands

**CCCC** Collection of Chen Changchin, Taiwan, China

**CBWX** Collection of BI Wenxuan, Shanghai, China

Male genitalia and female reproductive organs of specimens were extensively dissected. The dissected process follows Yang et al. (2013), and female membranous parts of reproductive organs were dyed with Chlorazol Black. Habitus images were captured using a Nikon D7000 digital camera with a Tamron SP 90mm lens. Terminalia images were captured by a Canon 450D digital camera fitted to a Nikon SMZ–1500 stereoscopic dissecting microscope.

Morphological terminology follows the works of Ekis (1977) and Opitz (2010) in general. For the convenience of taxonomic description and phylogenetic analysis, elytral asetiferous punctations are classified into primary asetiferous punctation (**PAP**) and accessory asetiferous punctation (**AAP**). PAP refers to the major ten rows of punctations which are also present in many other genera of Tillinae, such as *Tillus*, *Cladiscus*, *Diplopherusa*, and *Diplocladus* (Fig. 10B, C). AAP is the additional punctation that presents on interspaces among PAP rows and, in *Gastrocentrum* specifically, AAP presents on interspaces between 1^st^–2^nd^, 3^rd^–4^th^, and 5^th^–6^th^PAP rows (Fig. 10B, a). In some species such as *G. zayuense*, PAP decreases in quantity and PAP rows are less than ten rows.

The term microtrichia on the inner surface of elytra was adopted from Gorb (2001). A new term interphallic plate was introduced to depict a plate that inserted at the membranous part of phallus where situated between the two phallic plates (Fig. 11C, H, ipp).

### Phylogenetic analysis

Phylogenetic analysis was made using PAUP 4.0a (build 167) (Swofford 2002). Twenty-two morphological characters for eight ingroup and two outgroup taxa were compiled and analyzed. *Gastrocentrum brevicolle* was not included. Both the two species of *Isocymatodera* (*I. kolbei* and *I. atricolor*) were selected as outgroups. Exheuristic maximum parsimony analyses were performed. Characters were unordered and of equal weight. Branch support was determined for parsimony analyses using bootstrap with 1000 replicates in PAUP*. A bootstrap consensus tree and a list of character changes were obtained by PAUP*, and unambiguous character were mapped onto the tree by Illustrator 21.0.0.

Morphological characters used in the phylogenetic analysis are listed below. All the characters were coded as binary. Unknown or not applicable data coded as “?”. The data matrix is given in Table 1.

**Table 1.** Morphological character matrix used in estimation of phylogeny.

	1	2	3	4	5	6	7	8	9	10	11	12	13	14	15	16	17	18	19	20	21	22
*I. kolbei*	1	1	1	0	1	0	0	0	1	1	1	0	1	0	0	0	1	0	1	1	1	1
*I. atricolor*	1	1	1	0	1	0	0	0	1	1	1	0	1	0	0	0	1	0	1	1	1	0
*G. unicolor*	0	0	0	1	1	1	1	1	1	1	0	1	1	1	1	0	0	1	1	1	1	0
*G. laterimaculatum*	0	0	?	1	1	1	1	0	1	1	0	1	1	1	1	0	?	?	?	?	?	?
*G. magnum*	0	1	1	1	1	1	0	1	1	0	0	1	1	1	1	1	0	1	1	1	0	1
*G. dux*	0	?	1	1	1	1	0	1	1	0	0	1	0	?	?	?	0	1	0	1	0	0
*G. regulare*	0	1	1	1	0	0	0	1	1	0	0	1	1	1	1	0	0	1	1	0	0	0
*G. xiaodongi*	0	?	0	0	0	0	0	1	1	0	0	1	0	?	?	?	0	1	1	0	0	0
*G. zayuense*	0	0	1	0	0	0	0	1	0	0	0	1	0	1	0	1	0	1	1	0	0	0
*G. gaoligongense*	0	0	1	1	0	0	0	1	0	0	0	1	0	1	0	1	0	0	1	0	0	0

**Figures 1–9.**
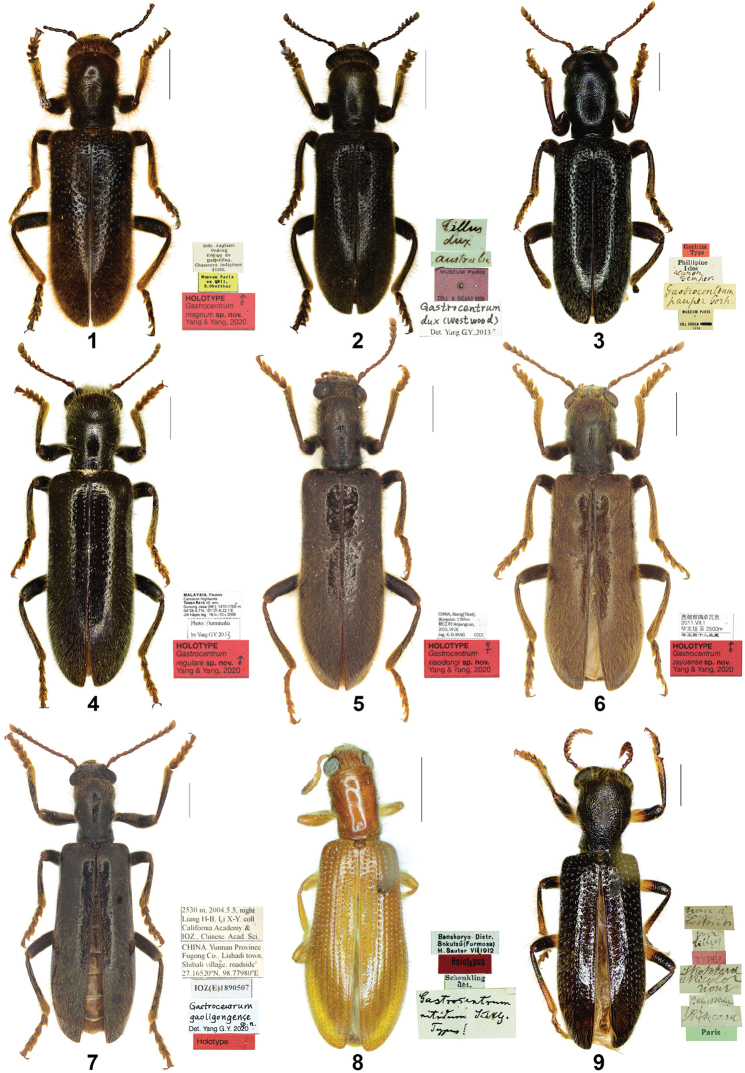
Habitus. **1***Gastrocentrum magnum* sp. nov. Holotype **2***G. dux* from Australia **3***G. unicolor* (Lectotype of *G. pauper*) **4***G. regulare* sp. nov. Holotype **5***G. xiaodongi* sp. nov. Holotype **6***G. zayuense* sp. nov. Holotype **7***G. gaoligongense* sp. nov. Holotype **8***Tillus nitidus* comb. nov. Holotype **9***Isocymatodera atricolor* Syntype. Scale bars: 5mm (**1, 2**) 2mm (**3–9**).

**Figure 10.**
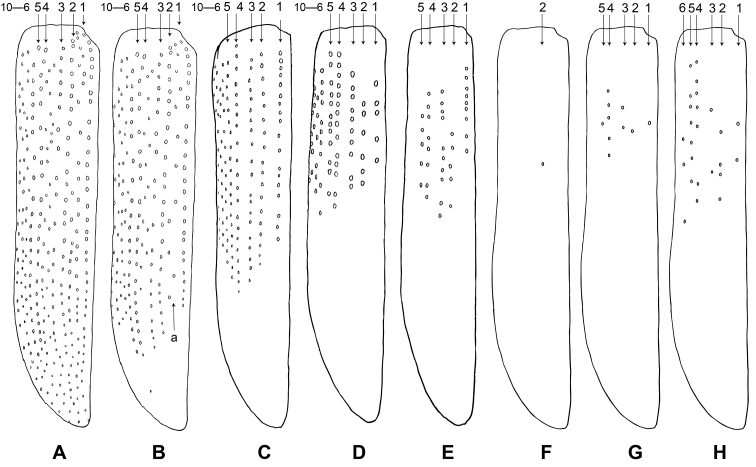
Drawing of elytral asetiferous punctations. **A***Gastrocentrum magnum* sp. nov. **B***G. dux***C***G. regulare* sp. nov. **D***G. xiaodongi* sp. nov. **E***G. gaoligongense* sp. nov. **F–H***G. zayuense* sp. nov. The numbers 1–10 annotate the serial rows of primary asetiferous punctuations (PAP). Abbreviations: **a** accessory asetiferous punctations.

**Figure 11.**
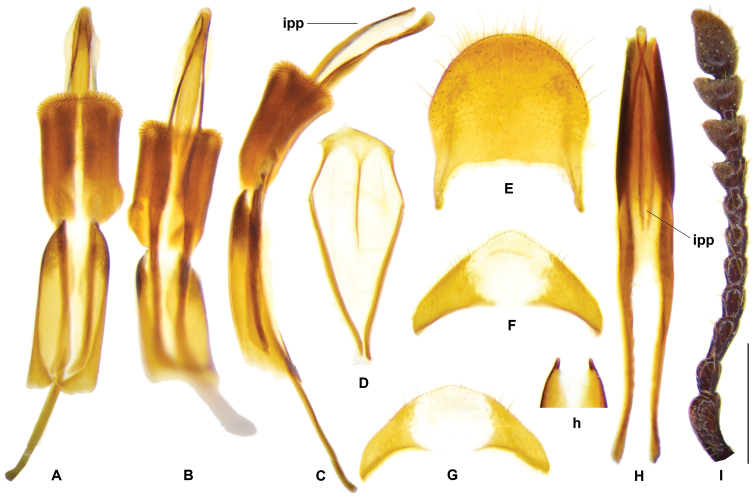
*Gastrocentrum unicolor*. **A–F** Lectotype of *G. pauper*. **A** aedeagus in dorsal view **B** aedeagus in ventral view **C** aedeagus in lateral view **D** spicular fork **E** pygidium **F** sixth ventrite. **G, H** specimen from Sulawesi. **G** sixth ventrite **H** phallus enveloped by connecting membrane, ventral view **h** apex of tegmen, ventral view **I** antenna. Abbreviations: **ipp** interphallic plate. Scale bar: 1 mm.

**Figure 12.**
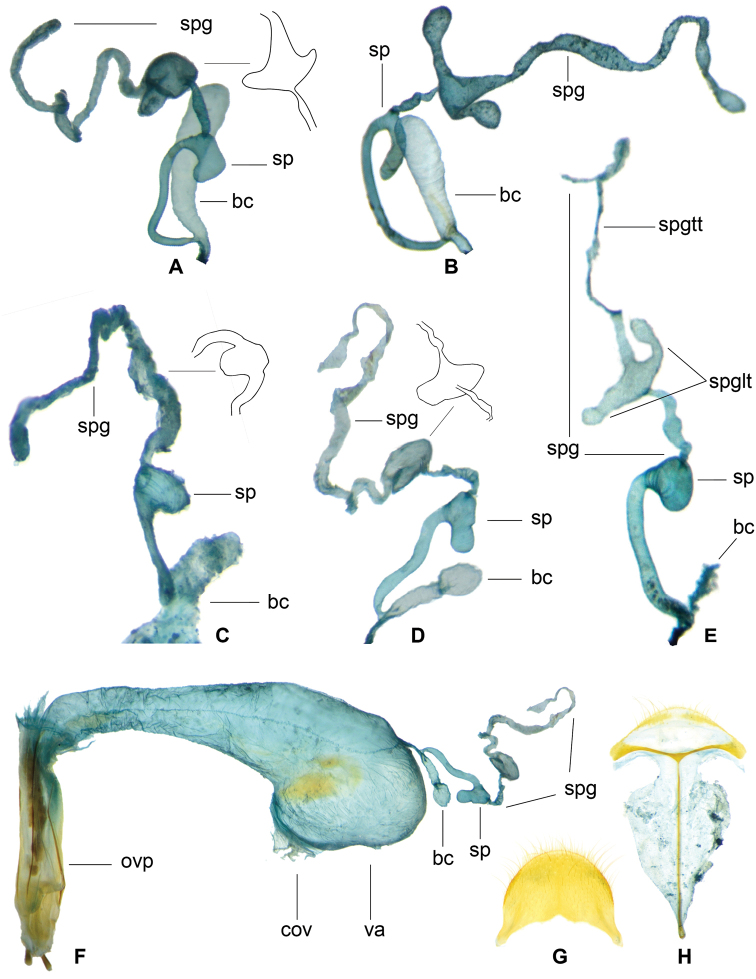
*Gastrocentrum unicolor* female reproductive organs specimens from different localities. **A** from Laos **B** from Hainan **C** from Philippines, Lectotype of *G. pauper***D** from Borneo **E** from Sulawesi **F** from Borneo **G** pygidium **H** sixth ventrite. Abbreviations: **bc** bursa copulatrix **cov** common oviduct i.e. median oviduct **ovp** ovipositor **sp** spermatheca **spg** spermathecal gland **spgtt** top tail of spermathecal gland **spglt** lateral tail of spermathecal gland **va** vagina.

**Figure 13.**
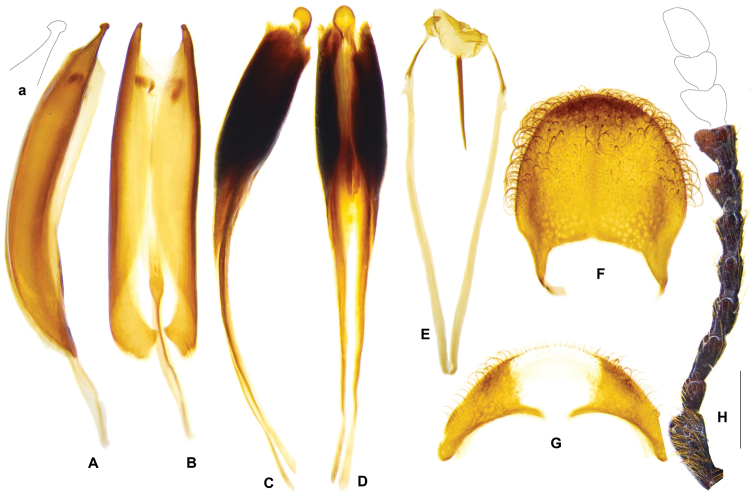
*Gastrocentrum magnum* sp. nov. Holotype. **A** tegmen in lateral view **a** apex of tegmen in lateral view **B** tegmen in ventral view **C** phallus in lateral view **D** phallus in ventral view **E** spicular fork **F** pygidium **G** sixth ventrite **H** right antenna lacking last three segments. Scale bar: 1 mm.

**Figure 14.**
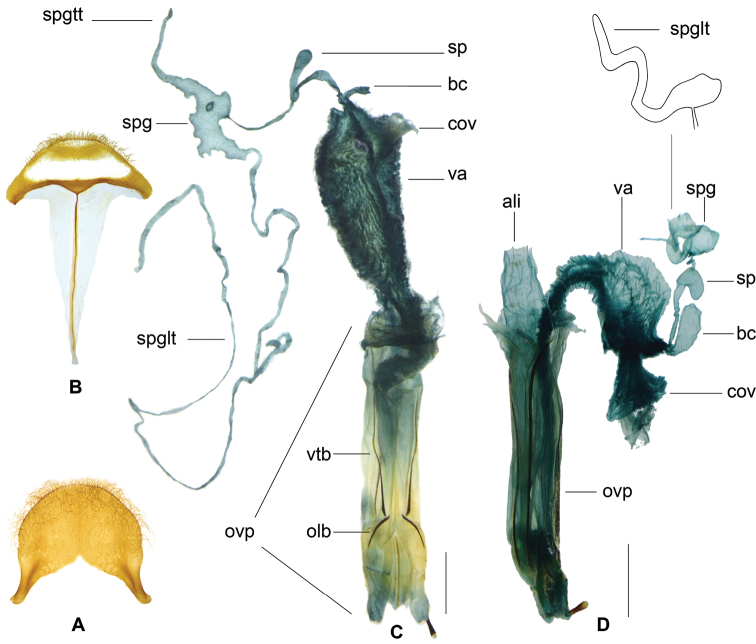
**A–C***Gastrocentrum magnum* sp. nov. female, paratype from Assam **A** pygidium **B** sixth ventrite **C** female reproductive organ **D***Gastrocentrum dux* from Australia, female reproductive organ. Abbreviations: **ali** alimentary canal **bc** bursa copulatrix **cov** common oviduct **olb** oblique bacculi **ovp** ovipositor **sp** spermatheca **spg** spermathecal gland **spgtt** top tail of spermathecal gland **spglt** lateral tail of spermathecal gland **va** vagina **vtb** ventral bacculi. Scale bar: 1 mm.

**Figure 15.**
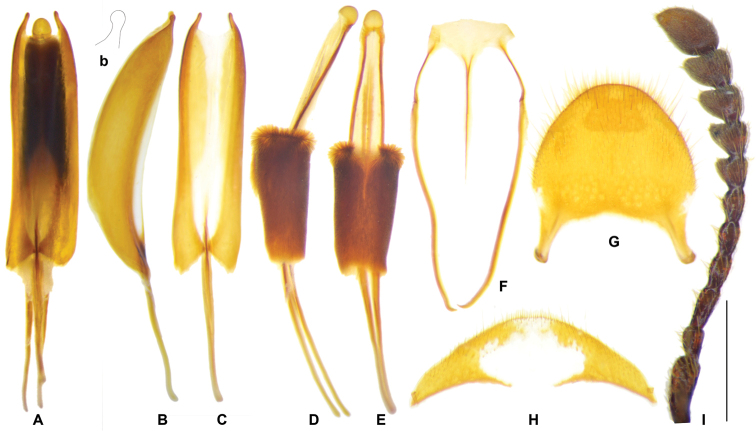
*Gastrocentrum regulare* sp. nov. male Holotype. **A** aedeagus in ventral view **B** tegmen in lateral view **b** apex of tegmen in lateral view **C** tegmen in ventral view **D** phallus with connecting membrane inverted, lateral view **E** phallus with connecting membrane inverted, ventral view **F** spicular fork **G** pygidium **H** sixth ventrite **I** antenna. Scale bar: 1 mm.

**Figure 16.**
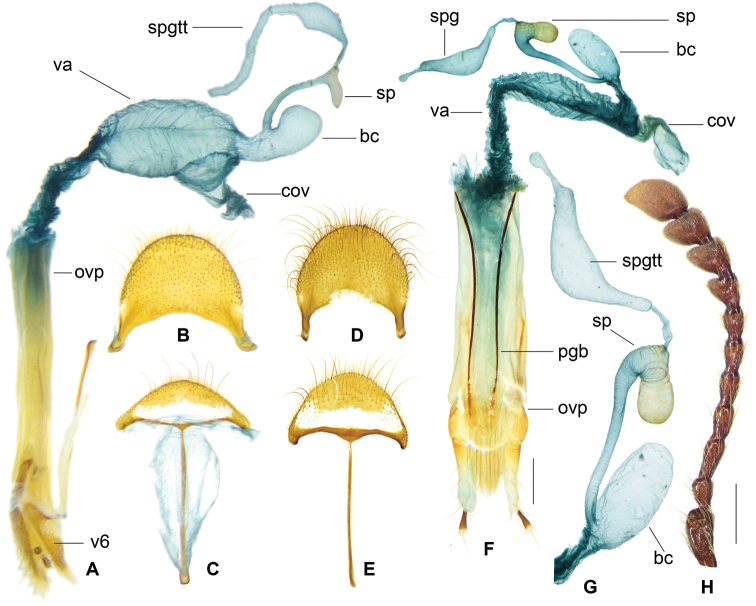
**A–C***Gastrocentrum regulare* sp. nov. female paratype **A** female reproductive organ **B** pygidium **C** sixth ventrite **D–H***Gastrocentrum xiaodongi* sp. nov. female holotype **D** pygidium **E** sixth ventrite **F, G** female reproductive organ **H** antenna. Abbreviations: **bc** bursa copulatrix **cov** common oviduct **ovp** ovipositor **pgb** proctigeral bacculi **sp** spermatheca **spg** spermathecal gland **spgtt** top tail of spermathecal gland **va** vagina **v6** sixth ventrite. Scale bars: 0.5mm (**A–F, H**).

**Figure 17.**
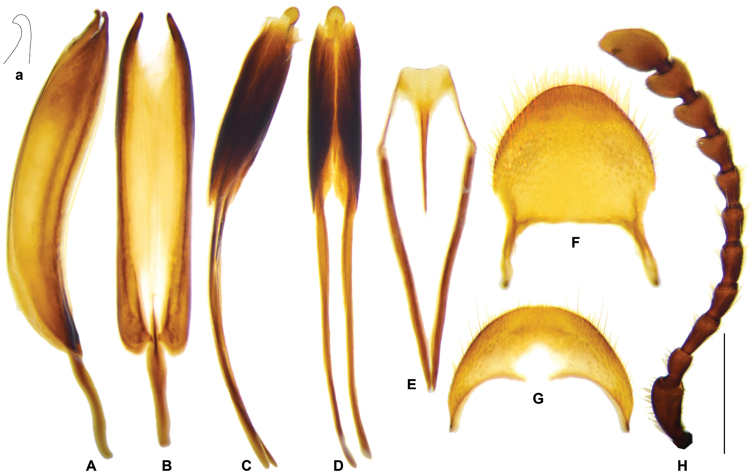
*Gastrocentrum zayuense* sp. nov. Holotype male. **A** tegmen in lateral view **a** apex of tegmen in lateral view **B** tegmen in ventral view **C** phallus in lateral view **D** phallus in ventral view **E** spicular fork **F** pygidium **G** sixth ventrite **H** antenna. Scale bar: 1mm.

**Figure 18.**
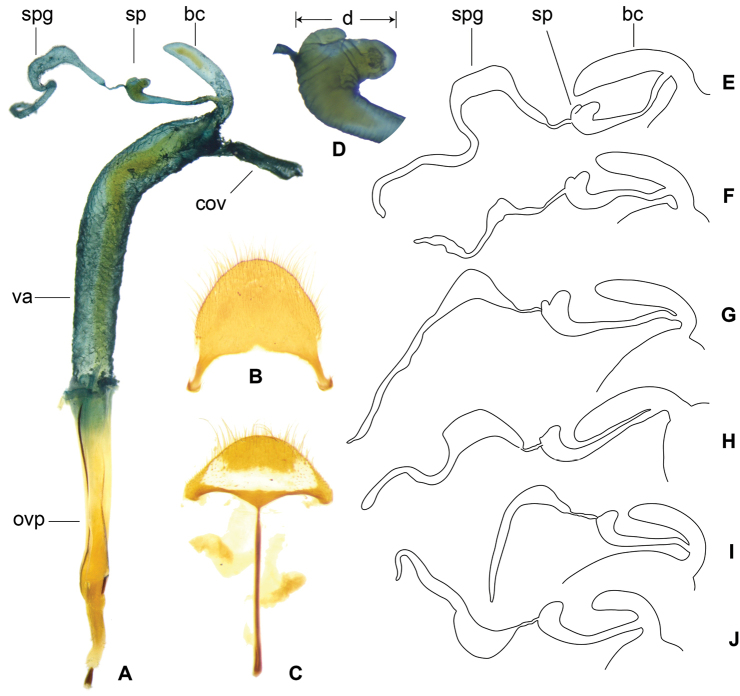
*Gastrocentrum zayuense* sp. nov. paratypes, female reproductive organs of different specimens showing morphological variations. **A** female reproductive organ **B** pygidium **C** sixth ventrite **D** spermatheca **E–J** drawings of bursa copulatrix, spermatheca and spermathecal gland of six females. Abbreviations: **bc** bursa copulatrix **cov** common oviduct d distal part of spermathecal capsule **ovp** ovipositor sp spermatheca **spg** spermathecal gland **spgtt** top tail of spermathecal gland **spglt** lateral tail of spermathecal gland **va** vagina.

**Figure 19.**
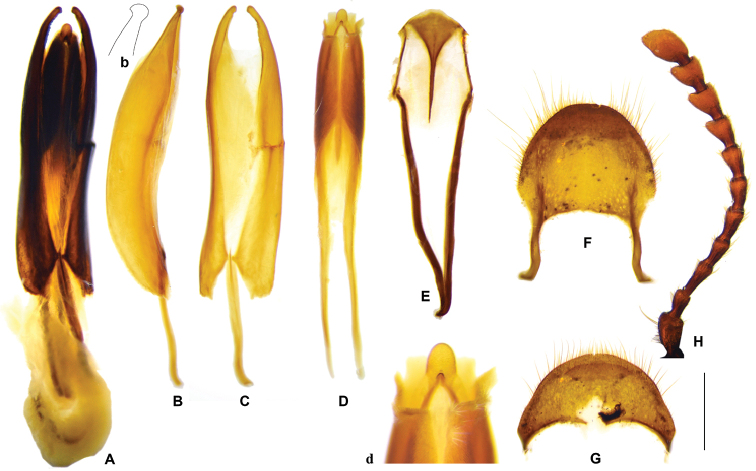
*Gastrocentrum gaoligongense* sp. nov. Holotype male. **A** aedeagus in ventral view **B**. tegmen in lateral view **b** apex of tegmen in lateral view **C** tegmen in ventral view **D** phallus in ventral view **d** apex of phallus **E** spicular fork **F** pygidium **G** sixth ventrite **H** antenna. Scale bars: 1mm.

**Figures 20–21.**
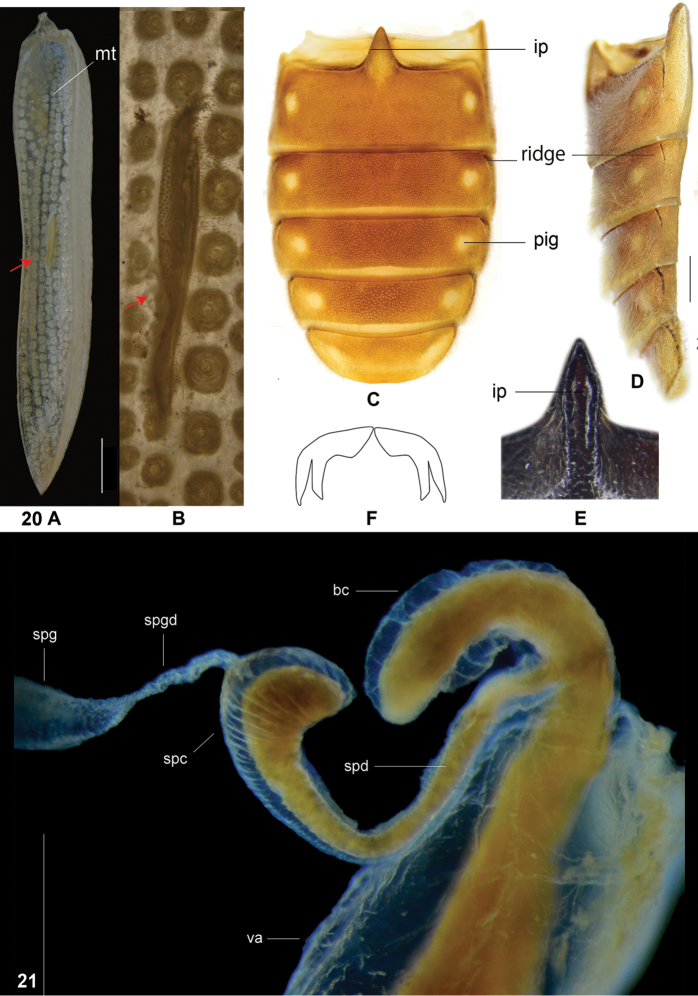
**20A, B** wedge-shaped protuberance on inner surface of elytron with *G. unicolor* as an example **C, D** abdomen of *G. zayuense* in ventral and lateral view showing 1^st^–5^th^ ventrites with short lateral ridges on each segments **E** intercoxal process of first ventrite of *G. unicolor* showing the longitudinal groove **F** claws of *Isocymatodera atricolor*. **21** Female reproductive organs of *G. zayuense* sp. nov. not stained and with the dark background, revealing the internal tissues. Abbreviations: **bc** bursa copulatrix **ip** intercoxal process **mt** microtrichia field **pig** less pigmented circles on abdominal segments **ridge** short ridges on abdominal segments **spc** spermathecal capsule **spd** spermathecal duct **spg** spermathecal gland **spgd** spermathecal gland duct **va** vagina. Scale bars: 0.5 mm (**20A–D**) 1 mm (**21**).

**Figure 22.**
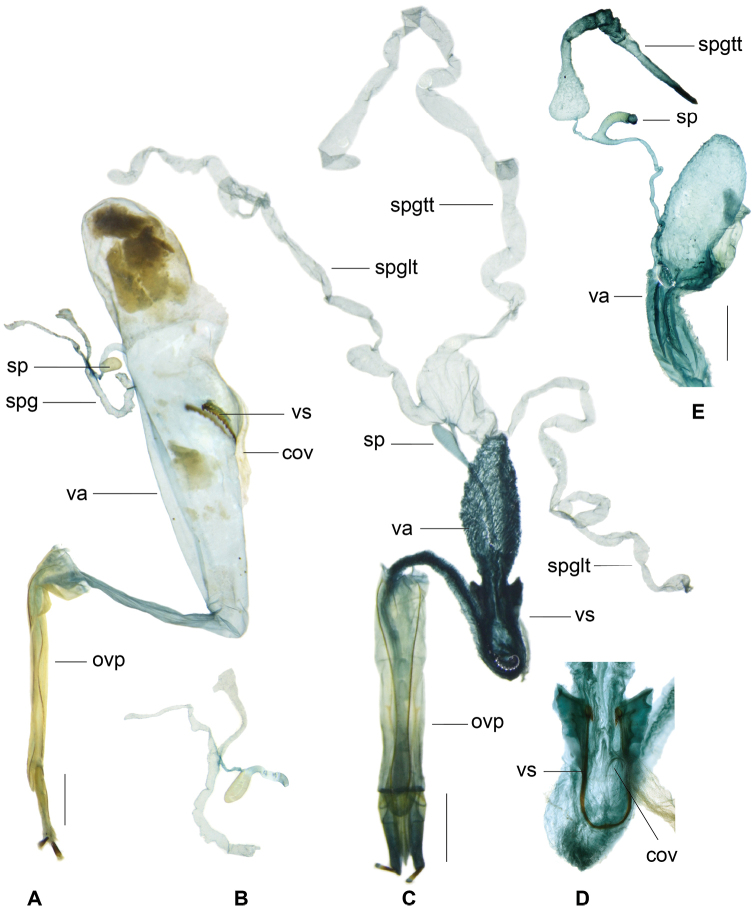
**A, B***Isocymatodera atricolor*. **C, D***I. kolbei*. **E***Gastrocentrum gaoligongense*. Abbreviations: **cov** common oviduct **ovp** ovipositor **sp** spermatheca **spg** spermathecal gland **spgtt** top tail of spermathecal gland **spglt** lateral tail of spermathecal gland **va** vagina **vs** vaginal sclerite. Scale bar: 1 mm.

1. 4 th antennomeres serrate, extended laterally: (0) no; (1) yes.
2. Male 7 th antennomeres broadly extended laterally: (0) no (Fig. 11I); (1) yes (Fig. 15I).
3. Female 7 th antennomeres broadly extended laterally: (0) no; (1) yes.
4. Elytral inner surface with wedge-shaped protuberance: (0) no; (1) yes (Fig. 20A, B).
5. Elytral interspace between 1 st2 nd PAP rows possesses AAP: (0) no (Fig. 10C–H); (1) yes (Fig. 10A, B).
6. Elytral interspace between 3 rd–4 thand 5 th–6 th PAP rows possesses AAP: (0) no (Fig. 10C–H); (1) yes (Fig. 10A, B).
7. Elytral AAP distinctly arranged in two rows: (0) no; (1) yes.
8. Distance between 2 nd-3 rd PAP rows greater than diameter of PAP: (0) no; (1) yes (Fig. 10A–H).
9. Elytral punctations reach lateral margins: (0) no (Fig. 10E–H); (1) yes (Fig. 10A–D).
10. Protibial outer-apical tooth present: (0) absent; (1) present (Gerstmeier 1993: fig. 2).
11. Mesotibial outer-apical tooth present: (0) absent; (1) present (Gerstmeier 1993: fig. 2).
12. Abdomen with lateral ridge on 1 st–5 th segments: (0) no; (1) yes (Fig. 20C, D, ridge).
13. Intercoxal process of first abdominal ventrite grooved longitudinally: (0) no (Fig. 20C, ip); (1) yes (Fig. 20E, ip).
14. The micro-hooked connecting membrane of male aedeagus extended to ventral surface: (0) no (Fig. 17G); (1) yes (Fig. 15D, E).
15. Male 6 th ventrite with membranous region extending to posterior margin: (0) no; (1) yes (Fig. 11F, G)
16. Male tegmen apices hooked: (0) no (Figs 11h, 15b); (1) yes (Figs 13A, 17A, 19B).
17. Female vagina with sclerites: (0) no (Figs 12F, 16A); (1) yes (Fig. 22A, C, D, E).
18. Female bursa copulatrix clearly defined: (0) no (Fig. 22A, C, E); (1) yes (Figs 12F, 16A, 18A, 21).
19. Female spermathecal gland with a top tail: (0) no (Fig. 14D); (1) yes (Figs 12E, 14C, 16A, F, 18A, 22A, 22C).
20. Female spermathecal gland with one or more lateral tails: (0) no (Figs 16A, F, 18A, 22E); (1) yes (Figs 12E, 14C, D, 22A, C).
21. Female spermathecal gland with two lateral tails: (0) no (Figs 14C, D, 16A, F, 18A, 22E); (1) yes (Figs 12E, 22B, C).
22. Female spermathecal gland with any of the tail extremely long, much longer than ovipositor: (0) no (Fig. 16A, F); (1) yes (Figs 14C, 22C).

## Results

### Taxonomic accounts

#### Genus Gastrocentrum

Taxon classificationAnimaliaColeopteraCleridae

Gorham, 1876

451565E1-1D67-5B60-ABF4-55DB333AD2AB

Gastrocentrum Gorham, 1876: 63 (Type species: Gastrocentrum pauper Gorham, 1876; by original designation); Chapin, 1924: 166, 179 (redescription).
Exocentrum Pic, 1940: 3 (printer error).

##### Diagnosis.

The genus *Gastrocentrum* was included in the *Philocalus* genus group close to the genus *Isocymatodera* (Gerstmeier 2005; Gerstmeier and Weiss 2009). Both genera have the claw with one inner denticle, which is similar to and only very slightly smaller than the apical portion of the claw (Fig. 20F). The genus *Gastrocentrum* can be differentiated from *Isocymatodera* by antennae broadly expanded laterally from 7^th^ or 8^th^ antennomeres onwards, the connecting membrane of male aedeagus specialized in both dorsal and ventral surface, the female vagina devoid of sclerites. While in *Isocymatodera*, the antennae are expanded laterally from 3^rd^ or 4^th^ antennomeres onwards, the connecting membrane of male aedeagus is only specialized in dorsal surface, and the female vagina possesses sclerites.

##### Redescription.

***General appearance*:** body length 9–29 mm; oblong, somewhat robust; all the species except *G. laterimaculatum* uniformly dark brown (Figs 1–9); vested with long, yellow setae all over the body. ***Head***: hypognathous, moderately large, including eyes slightly broader than pronotum; eyes sizable, emarginate, coarsely faceted, ocular notch small, distance of eyes as long as or only slightly greater than transverse diameter of eyes; gula broad, gular sutures parallel or slightly converging in anterior; antenna comprised of eleven antennomeres, broadly expanded laterally from 7^th^ or 8^th^–11^th^, the expanded antennomeres triangular except the last one cultriform, all the expanded antennomeres clothed with fine and dense pubescence; labrum emarginate, mandibles stout with inner dens, terminal segment of maxillary palpi digitiform, that of labial palpi broadly securiform. ***Thorax***: pronotum long campaniform, constricted posteriorly, anterior transverse depression feeble, surface punctate, faintly wrinkled, clothed with long, yellow hairs; pro-intercoxal process thin. ***Elytra***: oblong, sides parallel, anterior ridge present from humerus to scutellum; inner surface with a wedge-shaped protuberance at lateral middle of each elytron (Fig. 20A, B), leaning to the lateral side of first ventrite of abdomen in resting position (except for two species: *G. xiaodongi* and *G. zayuense*), and with a microtrichia field on anterio-lateral area (Fig. 20A, mt; similar to the structure found in Tenebrionidae, Gorb, 2001: 125, fig. 8.1, EAL); elytra have two types of punctations: asetiferous and setiferous punctations, the former comprised of primary asetiferous punctation (PAP) and accessory asetiferous punctation (AAP); each elytron possesses ten rows of PAP in general, the fifth row situate just before the humerus (Fig. 10C, D), sometimes PAP vanish in lateral elytron (in *G. zayuense* and *G. gaoligongense*, Fig. 10E–H); AAP may present on the interspaces between 1^st^–2^nd^, 3^rd^–4^th^, and 5^th^–6^th^PAP rows and similar with PAP in size, making these two types of punctations more or less indistinguishable (Fig. 10A, B); setiferous punctations minute, bearing setae, densely dispersed over the whole elytral surface, not in rows. ***Legs***: tibia without longitudinal ridge, tibial spur formula 1–2–2 (but in *G. laterimaculatum* 0–2–2), protibia without tooth at outer apex (except in *G. unicolor* and *G. laterimaculatum* where protibia possess a blunt tooth at outer apex); tarsi formula 5–5–5, first to fourth tarsomeres of all legs more or less bilobed and bearing evident pulvilli; claw with one inner denticle, which similar to and only very slightly smaller than apical portion of claw (Fig. 20F). ***Abdomen***: abdomen longer than broad, parallel in front and tapering in rear; first to fifth abdominal ventrites each with a pair of short longitudinal ridges (Fig. 20D) and a pair of less pigmented circles (Fig. 20C) in lateral; sixth ventrite partly or totally slid under the fifth, so in parts of specimens only five ventrites visible; intercoxal process of the first ventrite keel-like, with or without longitudinal groove; first ventrite strongly ridged behind metacoxae. ***Male genitalia***: pygidium subquadrate; sixth ventrite subtriangular to semicircle, posterior margin somewhat rounded, secondary sexual modifications slight (Figs 11F, G, 13G, 15H, 17G, 19G); central parts of sixth ventrite membranous, shape of membranous region different among species; spicular fork well developed, plates slender, apodemes not fused centrally, longitudinal intraspicular plate present (Figs 11D, 13E, 15F, 17E, 19E); tegmen tubiform, sclerotized from dorsal midline to lateral sides, barely sclerotized and unpigmented in ventral middle, tegmen lobed distally, parameres bent to ventral direction, tip simple or hooked, phallobasic apodeme present (Fig. 15B, C); phallus comprised of two thin phallic plates devoid of dentations, an interphallic plate present on the membrane between the two phallic plates (Fig. 11C, H, ipp), phallus apex simple, knot-like, phallic struts long and slender; connecting membrane between tegmen and phallus well sclerotized and thickened except the dorsal midline and ventral midline, forming a nearly whole sheath covering the phallus, which surface densely equipped with microhooks (Fig. 11A–C). ***Female reproductive organs***: pygidium subquadrate; sixth ventrite sub-triangular, disc membranous (Figs 12H, 14B, 16C, E, 18C), spiculum ventrale present; ovipositor as long as abdomen, moderately sclerotized, light yellow, semi-transparent (Figs 12F, 14C, D, 16A, F, 18A, ovp), with proctigeral bacculi in dorsal surface (Fig. 16F, pgb) and ventral and oblique bacculi in ventral surface (Fig. 14C, vtb, olb); vagina and alimentary canal partially enclosed in ovipositor, unenclosed part of vagina as long as or slightly longer than ovipositor, tubular or saccular; bursa copulatrix clearly defined and positioned distally (Figs 12F, 16A, 18A, bc) with the exception of the species *G. gaoligongense*, where bursa copulatrix is a mere swollen continuation of the vagina (Fig. 22E); spermatheca attached to the base of bursa copulatrix, boundary between spermathecal duct and spermathecal capsule somewhat obscure (Figs 16G, 21), spermathecal duct slender (Fig. 16A) or sometimes inflated in distal and continuous with spermatheca (Figs 16G, 18A), spermathecal capsule moderately to minimally sclerotized, both spermathecal duct and spermathecal capsule with spiral micro-texture, distal part of spermathecal capsule strongly bent, angled no more than 90° (Fig. 16A, G), spermathecal gland duct inserted at the outer edge of the angle (Fig. 21, spgd); in ground-plan, spermathecal gland have three tail-like endings: one located distally, opposite to its opening to spermatheca (top tail, Figs 12E, 14C, spgtt), the other two situated laterally (lateral tail, Figs 12E, 14C, spglt), any of which may be missing in different species and sometimes can be extremely long (Fig. 14C, spglt).

##### Distribution.

Indian subcontinent to Indochinese Peninsula and south through Malay Archipelago to Australia, including the following countries: India, Sri Lanka, Philippines, China, Vietnam, Laos, Malaysia, Indonesia, Australia (Fig. 25).

### Key to species of *Gastrocentrum* Gorham (not including *G. brevicolle*)

**Table:** 

1	Antennae expanded laterally from 3^rd^ or 4^th^ antennomere onwards	*** Isocymatodera ***
–	Antennae expanded laterally from 7^th^ (Fig. 15I) or 8^th^ (Fig. 11I) antennomere onwards	**2**
2	AAP present on elytral interspaces between 1^st^–2^nd^, 3^rd^–4^th^, and 5^th^–6^th^PAP rows, total punctations arranging in more than ten rows (Fig. 10A, B)	**3**
–	AAP absent on elytral interspaces between 1^st^–2^nd^, 3^rd^–4^th^, and 5^th^–6^th^PAP rows, total punctations arranging in ten or less rows (Fig. 10C–H)	**6**
3	Elytra uniformly brown; punctations smaller, with diameter smaller than interspace between 2^nd^ and 3^rd^PAP rows	**4**
–	Elytra yellow-brown; each elytron with a nearly semicircular large dark spot in lateral middle, elytral punctations larger, with diameter greater than interspace between 2^nd^–3^rd^ rows	***G. laterimaculatum***
4	Antennae broadly expanded laterally from 7^th^ antennomere onwards (Fig. 15I)	**5**
–	Antennae broadly expanded laterally from 8^th^ antennomere onwards (Fig. 11I)	***G. unicolor***
5	Elytral asetiferous punctations continuing to the tip (Fig. 10A), intercoxal process of first abdominal ventrite grooved longitudinally (Fig. 20E); female pygidium with a small triangular notch in anterior margin (Fig. 14A); top tail of spermathecal gland present (Fig. 14C, spgtt), lateral tail ca. 2× the length of ovipositor (Fig. 14C, spglt)	***G. magnum* sp. nov.**
–	Elytral asetiferous punctations stop by apical fifth, not continuing to the tip (Fig. 10B), intercoxal process of first abdominal ventrite not grooved longitudinally (Fig. 20C, ip); female pygidium with a large semi-circle notch in anterior margin; top tail of spermathecal gland absent, lateral tail much shorter than ovipositor (Fig. 14D, spglt)	***G. dux***
6	Elytral punctations reach lateral margin of elytra, arranging in ten rows (Fig. 10C, D)	**7**
–	Elytral punctations vanished at lateral sides of elytra, arranging in six rows at most (Fig. 10E–H)	**8**
7	7^th^ antennomere conspicuously expanded laterally (Fig. 15I); elytra asetiferous punctations stop by apical third (Fig. 10C); elytral inner surface with wedge-shaped protuberance (Fig. 20A, B); intercoxal process of first abdominal ventrite grooved longitudinally (Fig. 20F, ip); spermathecal capsule slender, tapering to the tip, length/width ratio = 3.6 (Fig. 16A, sp).	***G. regulare* sp. nov.**
–	7^th^ antennomeres barrel-shaped, not expanded laterally; elytra asetiferous punctations stop by middle (Fig. 10D); elytral inner surface without wedge-shaped protuberance; intercoxal process of first abdominal ventrite not grooved longitudinally (Fig. 20C, ip); spermathecal capsule thicker, rounded in apex, length/width ratio = 2.0 (Fig. 16F, sp).	***G. xiaodongi* sp. nov.**
8	Elytral inner surface without wedge-shaped protuberance; male tegmen apices with ventral surface streamlined (Fig. 17a); female bursa copulatrix clearly defined, much narrower than vagina, distal part of spermathecal capsule short, more or less inflated, length/width ratio < 2.5 (Fig. 18A)	***G. zayuense* sp. nov.**
–	Elytral inner surface with wedged-shaped protuberance (Fig. 20A, B); male tegmen apices with ventral surface bulged (Fig. 19b); female bursa copulatrix not differentiated, merely a swollen continuation of the vagina, distal part of spermathecal capsule long and slender, length/width ratio > 5 (Fig. 22E)	***G. gaoligongense* sp. nov.**

#### Gastrocentrum unicolor

Taxon classificationAnimaliaColeopteraCleridae

(White, 1849)

2271B5D0-3455-58CC-80A8-CD810C88BC2E

Figures 3 , 11 , 12 , 25

Notoxus unicolor White, 1849: 56 (type locality: “India”); Gahan, 1910: 61 (Gastrocentrum); Schenkling, 1912: 323 (Taiwan); Chapin, 1924: 179, pl. 1, f. 4 (Philippines); Corporaal, 1950: 55 (catalogue); Mawdsley, 1999: 270 (Sri Lanka).
Gastrocentrum pauper Gorham, 1876: 63 (type locality: “Luzon, Philippines”); Schenkling, 1903 (Dindigul, S. India); Gahan, 1910: 61 (synonymized with G. unicolor White).

##### Type specimens examined.

**Lectotype of *G. pauper*** designated herein (Fig. 3): “Gorham Type / Phillipine Isles, Luzon, Semper / Gastrocentrum pauper Gorh. [hw. by Gorham] / Museum Paris, Coll. Gorham, 1914” (MNHN, male, dissected); **Paralectotype of *G. pauper***: “Gorham Type / Camiguin de Luzon / Gastrocentrum genus novum, G. pauper Gorh. [hw. by Gorham] / Museum Paris, Coll. Gorham, 1914” (MNHN, 1 female, dissected).

##### Other specimens examined.

**China: Taiwan**: 1994-VII-30, Taiwan, Taoyuan County, Fuxing Township, Shang Baling, 1200 m (CCCC, 1 male, dissected); 2005-IX-4, Taiwan, Taidung County, Beinan Township, Lijia Forest Trail, 1300 m, W-I. Chou leg. (CCCC, 1 male, dissected); 11-IX-1996, Taiwan, Pingtung County, Kenting National Park, W. I. Chou leg. (CCCC, 1 male, dissected); Taiwan, Formosa, IV, Gastrocentrum unicolor White (pauper Gorh.), Museum Paris Coll. M. Pic (MNHN, 1 female); **Hainan**: Hainan, Wuzhi Mountain, 2011.IX.20, BI Wenxuan (CBWX, 1 female, dissected); Hainan Prov., Baisha, Nankai Town, on vegetation or ground, 18.9741°N, 109.2956°E, 790 m, 2010.4.13 D, Lin Meiying coll. (IZAS, 1 female, dissected). **Vietnam**: “Museum Paris, Tonkin N., Env. d’Ha-giang, Lieut. Col. Bonifacy 1913” (MNNH, 1 female, dissected). **Laos**: “Laos-NE, Houa Phan prov., 20°13'09–19"N 103°59'54"-104°00'03"E, 1480–1550 m, PHOU PANE Mt., 1.-16.vi.2009, Zdeněk Kraus leg./NHMB Basel, NMPC Prague, Laos 2009 Expedition: M. Brancucci, M. Geiser, Z. Kraus, D. Hauck, V. Kuban” (NHMB, 1 female, dissected). **Thailand**: N. Thailand, Meo Village, near Chiang Mai, V.1998 (RGCM, 1 ex.); Thailand, Corat, 26.III.1988 (RGCM, 1 ex.); Thailand, Chiangmai, Doi Pui, 12.VI.1985 (RGCM, 1 ex.). **Malaysia: Peninsular Malaysia**: Bukit Kutu, Selangor, April 1915, 3457 / ex. Coll. Zoologisch Museum Amsterdam (MNHN, 1 male, dissected); Malaysia, Pahang, Cameron Highlands, Tanah Rata vill. env., Gunung Jasar [Mt]; 1470–1705m, 04°28.4–7'N, 101°21.6–22.1'E, Jiří Hájek leg., 18.iv–10.v.2009 (NMPC, 1 female, dissected); **East Malaysia**: Elopura, N.-E. Borneo, W. B. Pryer. / Museum Paris ex Coll. R. Oberthur (MNHN, 2 males 1 female, dissected); Borneo, Sabah, Keningau district, Jungle Girl Camp. 5.4430°N, 116.4512°E; 1182 m; Shi H. L. & Liu Y. lgt. light trap, night, 2016.IV.25 (2 ex.), 2016.IV.29 (3 ex.), 2016.IV.30 (1 ex.), 2016.V.1 (1 ex.), 2016.V.2 (3 ex.) (FBFU). **Indonesia**: W. Celebes, G. Rangkoenau, J. P. Ch. Kalis, 900 ‘. 1937 (MNHN, 5 males, 4 females in total, of which 3 males, 4 females dissected); W. Celebes, Loda, Paloe, J.P. Ch. Kalis, 4000 ‘. 1937 (MNHN, 1 male, dissected); W. Celebes, Sjdaonta Paloe, J. P. Ch. Kalis, 4500’. 1937 (MNHN, 1 male, dissected); Bonthain, Celebes 8. ‘38 (MNHN, 1 male, dissected).

##### Diagnosis.

This species has the broadest distribution range in this genus. It is different from *G. magnum* sp. nov., *G. dux* sp. nov. and *G. regulare* sp. nov. in antennae broadly extended laterally from 8^th^ antennomere onwards (Fig. 11I); different from *G. xiaodongi* sp. nov., *G. zayuense* sp. nov., and *G. gaoligongense* sp. nov. in having AAP on interspaces between elytral 1^st^–2^nd^, 3^rd^–4^th^, and 5^th^–6^th^PAP rows.

##### Redescription

(based on type specimens of *G. pauper* and other specimens from SE Asia only). ***General appearance***: length 9–16 mm, oblong, robust, uniformly dark brown. ***Head***: including eyes feebly broader than pronotum; eyes moderately large, distance between eyes faintly larger than the transverse diameter of eye; gular suture parallel; antennae expanded laterally from 8^th^ antennomere onwards (Fig. 11I); vertex and frons densely punctate, postgenae rugose. ***Pronotum***: oblong, length/width ratio ca. 1.4, constricted posteriorly; surface densely punctate, faintly rugose, clothed with long, yellow hairs. ***Elytra***: oblong, sides subparallel, length/width ratio ca. 2.3, vested with dense light yellow or off-white setae; wedge-shaped protuberance present on inner surface (Fig. 20A, B); asetiferous punctations arranged in more than ten rows, PAP in ten rows, AAP on interspaces between 1^st^-2^nd^, 3^rd^-4^th^, and 5^th^-6^th^PAP rows, AAP setting in two rows on each interspace; AAP almost same size as PAP; interspace between 2^nd^-3^rd^PAP rows much wider than punctation diameter; both PAP and AAP beginning to decrease in size postmedially to apical third, and completely vanished at apical fourth to fifth. ***Legs***: outer apex of protibia extending outwards and forming a blunt tooth. ***Abdomen***: intercoxal process of the first ventrite conspicuously grooved longitudinally. ***Male genitalia***: pygidium subquadrate, posterior margin rounded (Fig. 11E); sixth ventrite sub-triangular, ca. 2 × as broad as long, posterior margin more or less angulate, central membranous region pentagonal or subquadrate, extending from anterior margin to posterior margin (Fig. 11F, G); tegmen with phallobasic apodeme 0.6 time as long as phallobase (Fig. 11A–C); paramere apices simple, petty, unhooked (Fig. 11A, h); interphallic plate slightly shorter than half length of phallus (Fig. 11C, H, ipp); phallus apex usually knot-like, 2~3 × as long as wide (Fig. 11H). ***Female reproductive organs***: pygidium slightly broader than long, posterior margin rounded (Fig. 12G); sixth ventrite 2.7 × broader than long, central membranous region elliptical, apical accessory membranous region petty (Fig. 12H); vagina swollen in well-preserved specimens, bursa copulatrix clearly defined (Fig. 12F), spermathecal gland with a top tail of medium length and two lateral tails that almost reduced (Fig. 12A–E); spermatheca boot-shaped in general (Fig. 12A, C–E).

##### Variation.

The tegmen apices of *G. unicolor* are simple, unhooked, unspecialized (Fig. 11A). However, specimens from Sulawesi with tegmen apices slightly more prominent than those from other regions. Phallus apex is normally knot-like with the two phallic plates convergent at a point before the tip (Fig. 11H), but in the holotype of *G. pauper*, edges of the two phallic plates are almost parallel to the tip (Fig. 11B). The apical tip of phallus is longer than broad, with length/width ratio varied in a range of 2.0~3.0; usually teardrop-shaped with length/width ratio 2.0~2.5, but oblong in specimens from Sulawesi with length/width ratio approximate to 3.0 (Fig. 11H).

Both of the two female specimens examined from Hainan has spermatheca tubiform (Fig. 12B), which is different from those from other localities with spermatheca inflated distally (Fig. 12A, C, D). However, given its same external structure and lacking male specimens, we consider the specimens from Hainan as the same species with *G. unicolor*.

##### Distribution.

This species is widespread, from Indian subcontinent to Indochinese Peninsula, south to Malay Archipelago, including the countries and regions: India, Sri Lanka, Philippines, China (Taiwan, Hainan), Vietnam, Laos, Thailand, Malaysia (Peninsular Malaysia, Sabah), Indonesia (Sulawesi).

##### Discussion.

Gahan (1910) proposed that *G. pauper* was a junior synonym of *G. unicolor* without explanation, which treatment was afterward followed by Schenkling (1912), Chapin (1924), Corporaal (1950), and temporarily by Mawdsley (1999) and the present paper. In our research, we have only examined specimens from SE Asia and determined they are identical with *G. pauper*. However, additional materials from India or Sri Lanka need to be compared with those from SE Asia thoroughly, which will lead to the confident assignment of the synonymy.

#### Gastrocentrum laterimaculatum

Taxon classificationAnimaliaColeopteraCleridae

Gerstmeier, 2005

FF834416-0EFC-5188-AE0A-333685FBBC9B

Figure 25

Gastrocentrum laterimaculatum Gerstmeier, 2005: 56 (type locality: Malaysia, Cameron Highlands).

##### Specimens examined.

**Malaysia**: H. C. Siebers, M. O. Borneo Exp. Long Hoet, 3.VIII.1925 (ZMAN, 1 ex.); Borneo, Sabah, Keningau district, Jungle Girl Camp., 5.4430°N, 116.4512°E, 1182m, Shi H. L. & Liu Y. lgt. light trap, 2016. V. 1. N (FBFU, 1 male); Malaysia, N. Borneo, Sabah, Keningau distr., Trus Madi Mt., h = 1160 m, leg. J. Chew, 20.VI.2011 (RGCM, 1 ex.).

##### Diagnosis.

This species is the only one in this genus that has elytral pattern and can be separated from other species without difficulty. Its elytra is yellow-brown, with a pair of large semicircular dark spots in lateral sides which is extended to the lower sides of humeri; elytral asetiferous punctations larger than other species, with punctation diameter greater than interspace between 2^nd^-3^rd^PAP rows; antennae broadly extended laterally from 8^th^ antennomere onwards.

##### Supplementary description.

Elytral wedge-shaped protuberance present on inner surface; elytral asetiferous punctations somewhat irregular, arranged in more than ten rows, PAP in ten rows, AAP on interspaces between 1^st^–2^nd^, 3^rd^–4^th^, and 5^th^–6^th^PAP rows, AAP setting in two rows on each interspace; AAP almost same size as PAP, which is more sizable than punctations in other species, with punctation diameter greater than interspace between 2^nd^–3^rd^PAP rows; both PAP and AAP beginning to decrease in size postmedially to apical third, and completely vanished at apical fifth; elytral inner surface with a wedge-shaped protuberance at lateral middle; tibiae spur formula 0–2–2 (other species in this genus 1–2–2); protibia with a blunt tooth at outer apex; 1^st^–2^nd^ abdominal ventrites dark brown, 3^rd^–5^th^ yellow, 6^th^ light yellow to transparent; first abdominal ventrite strongly ridged behind coxae, intercoxal process raised, triangular, slightly longer than broad, grooved longitudinally.

##### Distribution.

Malaysia (Peninsular Malaysia, Sabah).

#### Gastrocentrum magnum

Taxon classificationAnimaliaColeopteraCleridae

sp. nov.

4907A6E5-1E07-57BA-AC57-23B0F45B41F6

http://zoobank.org/111FBA09-A72D-401D-8178-8163A7F41C0F

Figures 1 , 10A , 13 , 14 , 25

##### Holotype.

**India**: “Inde Anglaise, Pedong, Région de Darjeeling. Chasseures indigènes, 1933 /Museum Paris, 1952, Coll. R. Oberthür / Gastrocentrum magnum sp. nov. males, Det. Yang G. Y. 2019 / Holotype: Gastrocentrum magnum sp. nov. Yang & Yang, 2020” (MNHN, male) (Fig. 1). **Paratypes**. **India**: “Assam / Museum Paris ex Coll. R. Oberthur” (MNHN, 1 male); “Assam, […] / Gastrocentrum dux Westw. / Museum Paris ex Coll. R. Oberthur / Ex-Musaeo H. W. Bates, 1892 / Museum Paris / females” (MNHN, 1 female); “Sikkim, Guntok, Eté 1894, Chasseurs Bretandeau / Gastrocentrum dux Westw. c.f. Gahan / Museum Paris ex Coll. R. Oberthur” (MNHN, 1 female). **China**: China, Xizang, Mêdog, Nyingchi, Baibung, 876 m, 2016.VIII.09, light trap, LU Yanquan leg. (CCCC, 1 female); Yunnan, Longchuan, 1770 m, 2016.VI.3, light trap, YANG Xiaodong leg. 16Y (CCCC, 1 female); “Hainan, Jianfengling, Tianchi, 2010.IV.15-20/Wenxin Lin, 950 m, Collection of CHEN Changchin” (CCCC, 1 female); China, Yunnan, Honghe, Lvshuihe, 640 m, 23°1'41"N, 103°24'19"E, 07.V.2019, leg. L.Z. Meng (NKME, 3 ex., RGCM,1 ex.); China, Yunnan, Honghe, Gulinqin, 585 m, 22°43'51"N, 103°59'35"E, 07.V.2019, leg. L.Z. Meng (RGCM, 2 ex.); China, S-Yunnan, Xishuangbanna, 20 km NW Jinghong, Man Dian NNNR-office, 22°07.80N, 100°40.05E, 740 m, LFF, 24.V.2008, leg. A. Weigel (RGCM, 1 ex.). **Vietnam**: C-Vietnam, ThuaThien – Hue Pr., Phu Loc, Bach Ma NP, Top area, 1250–1400 m, 16°11'39"N, 107°51'12"E, 5–9.V.2019, leg. A. Weigel LFF (RGCM, 1 ex.). **Thailand**: 18.–23.4.1991, Dol Suthep Pui, 1300–1500 m, leg. P. Pacholatko (RGCM, 1 male).

##### Diagnosis.

Earlier researchers identified one of the paratypes of this new species as *G. dux*. The new species can be separated from *G. dux* by: asetiferous punctations on elytra continuing to the tip (Fig. 10A); intercoxal process of first abdominal ventrite grooved longitudinally (Fig. 20F); female pygidium with anterior margin notched in a shallow triangular shape (Fig. 14A), and lateral tails of spermathecal gland extremely long, ca. 2 × the length of ovipositor (Fig. 14C, spglt). *Gastrocentrum unicolor* is sympatric with the new species in India, but *G. magnum* can be separated from it by much larger body size and five expanded terminal antennomeres (Fig. 13H).

##### Description.

***General appearance*:** length 22–25 mm, robust, dark brown. ***Head***: including eyes feebly broader than pronotum; eyes moderately large, distance between eyes slightly greater than the transverse diameter of eye; gular suture convergent in anterior; antennae expanded laterally from 7^th^ antennomere onwards (Fig. 13H); vertex and frons with dense punctations, with a very faint ridge along the midline, postgenae rugose. ***Pronotum***: oblong, length/width ratio ca. 1.5, constricted posteriorly; surface finely and densely punctate, faintly rugose, clothed with long, yellow hairs. ***Elytra***: oblong, sides subparallel, length/width ratio ca. 2.4, vested with dense golden setae; wedge-shaped protuberance present on inner surface; asetiferous punctations rows somewhat irregular, PAP in ten rows, AAP on interspaces between 1^st^–2^nd^, 3^rd^–4^th^, and 5^th^–6^th^PAP rows; AAP present in two incomplete rows, with longitudinal spacing between neighboring punctations uneven; AAP faintly smaller than or as big as PAP, interspace between 2^nd^–3^rd^PAP rows larger than punctation diameter (Fig. 10A); both PAP and AAP beginning to decrease in size postmedially and continuing to the tip, which are quite irregular near apical 1/5 of elytra (Fig. 10A). ***Legs***: outer apex of protibia not extending outwards. ***Abdomen***: intercoxal process of the first ventrite grooved longitudinally. ***Male genitalia***: pygidium subquadrate, posterior margin rounded (Fig. 13F); sixth ventrite arciform, 3 × wider than length, posterior margin well rounded, central membranous region inverted trapezoidal, extending from anterior margin to posterior margin (Fig. 13G); tegmen tubiform, length ratio of phallobasic apodeme to phallobase ca. 1: 3.7 (Fig. 13A, B); parameres hooked (Fig. 13A, a); interphallic plate shorter than half length of phallus (Fig. 13D); phallus apex knot-like, faintly longer than broad (Fig. 13C, D). ***Female reproductive organs***: pygidium slightly wider than length, posterior margin rounded (Fig. 14A); sixth ventrite trapezoidal, 3 × wider than long, posterior margin truncated, central membranous region broad, apical accessory membranous region petty (Fig. 14B); bursa copulatrix clearly defined; spermathecal gland with a short top tail (Fig. 14C, spgtt) and an extremely long lateral tail, which longer than twice length of ovipositor (Fig. 14C, spglt); spermatheca curved tubiform (Fig. 14C, sp).

##### Variation.

The female paratype collected from Hainan has the spermatheca faintly bifurcated distally. This individual variation was also observed in specimens of *G. zayuense* collected from the same locality (Fig. 18D–J).

##### Distribution.

India (Assam, Sikkim), China (Xizang, Yunnan, Hainan), Vietnam, Thailand.

##### Etymology.

This new species, together with *G. dux*, have the largest body size in this genus. The specific epithet comes from the Latin adjective *magnus* (=large).

#### Gastrocentrum dux

Taxon classificationAnimaliaColeopteraCleridae

(Westwood, 1852)

6369AC00-BB1A-55D9-9AB9-B04057A32622

Figures 2 , 10B , 14D , 25

Tillus dux Westwood, 1852: 46, pl. 24, f. 11 (type locality: “Nova Hollandia apud Fluvium Cygnorum”, = Australia, Swan River); Blackburn, 1900: 119 (Tillus); Gahan, 1910: 61 (Gastrocentrum); Corporaal, 1950: 55 (catalogue; “Ceylon, India, Laos, Java?, Australia??”); Mawdsley, 1999: 270 (Sri Lanka).

##### Specimens examined.

Australia: “Tillus dux / australie / Museum Paris, Coll. A. Sicard 1930 / Gastrocentrum dux (Westwood, 1852), Det. Yang G. Y. 2013” (MNHN, 1 female, dissected; Fig. 2).

##### Diagnosis.

The specimen examined can be separated with *G. magnum* by elytral asetiferous punctations stop by apical fifth, not continuing to the tip (Fig. 10B), intercoxal process of first abdominal ventrite not grooved, female pygidium with a semi-circle membranous region proximally, reaching half length of pygidium, lateral tail of spermathecal gland much shorter, only slightly longer than spermatheca (Fig. 14D, spglt).

##### Description.

***General appearance*:** length 23–29 mm, robust, dark brown. ***Head***: including eyes feebly broader than pronotum; eyes moderately large, distance between eyes nearly as long as the transverse diameter of eye; gular suture slightly convergent in anterior; antennae expanded laterally from 7^th^ antennomere onwards; vertex and frons densely punctate, with a very faint ridge along midline, postgenae rugose. ***Pronotum***: oblong, length/width ratio ca. 1.4, constricted posteriorly; surface finely and densely punctate, clothed with light yellow hairs. ***Elytra***: oblong, sides subparallel, length/width ratio ca. 2.31, vested with light yellow setae; wedge-shaped protuberance present on inner surface; PAP in ten rows, AAP on interspaces between 1^st^–2^nd^, 3^rd^–4^th^, and 5^th^–6^th^PAP rows; AAP present in two very incomplete rows, number of AAP less than that in *G. magnum*; AAP faintly smaller than PAP; interspace between 2^nd^–3^rd^PAP rows greater than punctation diameter; elytral punctations decreasing in size postmedially, and completely vanished at apical fifth (Fig. 10B). ***Legs***: outer apex of protibia very faintly extending outwards, not forming a distinct tooth. ***Abdomen***: intercoxal process of the first ventrite flat, not grooved. ***Male genitalia***: not studied. ***Female reproductive organs***: pygidium slightly wider than long, posterior margin rounded, a semi-circle membranous region present proximally, reaching to half length of the pygidium; sixth ventrite trapezoidal, wider than long; bursa copulatrix clearly defined; spermathecal gland only with one lateral tail, which slightly longer than spermatheca in fully stretched condition (Fig. 14D, spglt); spermatheca boot-shaped (Fig. 14D, sp).

##### Note on type specimen.

Mawdsley (1999) claimed that the type specimen of G. dux was deposited in the Hope Department of Entomology, University Museum, Oxford, United Kingdom, but it was not located during a visit to that museum in 2011 by the first author. Westwood (1852) indicated that the type specimen was from “Mus. Melly”, but efforts to locate it in Melly’s collection in the Natural History Museum, Geneva, yielded no results either. The whereabouts of the type specimen remains unknown.

##### Discussion.

The Australian type locality of this species is doubted by the Australian entomologist and clerid worker Justin Bartlett who, after viewing the Cleridae holdings of all major museum, and several agricultural and private collections from all Australian states, is yet to find a single *Gastrocentrum* specimen, and therefore does not believe *G. dux* to be an Australian species. He also doubts that the locality label of the specimen examined in this manuscript represents an actual collecting event, but rather was labelled after it was identified as *G. dux*, with the associated type locality of ‘Australie’ (pers. comm. J Bartlett). He also pointed out that another apparently Australian specimen from Melly’s collection, a longicorn *Hephaestion acraetus* Newman, is in fact a Chilean species (see Saunders 1850), providing a precedent for erroneously labelled specimens from Melly’s collection. Despite this, no more practical specimen-based evidence for or against this argument has been found. Hence, we can only describe this species based on the specimen mentioned above at the moment, as we can only take the label at face value and assume it to represent an actual collecting label.

We found a Tenebrionidae beetle with the same Swan River type locality also originating from Melly’s collection and described by Westwood: *Prophanes aculeatus* Westwood, 1849. It is presently treated as a valid species, with an eastern, not western, Australian distribution (Westwood 1849; Carter 1913; Matthews 1992). *Gastrocentrum dux* may have a similar historical story and its correct occurrence could be in other areas of Australia or in other regions of the world, but this hypothesis needs to be proved by further specimens.

#### Gastrocentrum regulare

Taxon classificationAnimaliaColeopteraCleridae

sp. nov.

790D18D7-2A83-5AAE-A728-6E6B03488F7E

http://zoobank.org/8463F3C7-F2A3-439E-AACD-0E238902A57A

Figures 4 , 10C , 15 , 16 , 25

##### Holotype.

Malaysia: “Malaysia, Pahang, Cameron Highlands, Tanah Rata vill. env., Gunung Jasar [Mt.]; 1470-1705m, 04°28.4–7'N, 101°21.6–22.1'E, Jiří Hájek leg. 18.iv–10.v.2009 / Holotype: Gastrocentrum regulare sp. nov. Yang & Yang, 2020” (NMPC, male, Fig. 4); **Paratype**. Same data as holotype (NMPC, 1 female).

##### Diagnosis.

This species is distinct in the genus in having ten regular rows of asetiferous punctations exceeding half of elytra, without AAP between the PAP rows. It can be differentiated from *G. xiaodongi* by: antennae expanded laterally from 7^th^ antennomere onwards (Fig. 15I); elytra punctations rows stop by apical third (Fig. 10C); inner surface of elytron with a wedge-shaped protuberance; intercoxal process of first abdominal ventrite grooved longitudinally; spermathecal capsule slenderer, tapering to the tip (Fig. 16A, sp).

##### Description.

***General appearance*:** length 12–14 mm, robust, dark brown. ***Head***: including eyes feebly broader than pronotum; eyes moderately large, distance between eyes almost as long as the transverse diameter of eye; gular suture almost straight-up; antennae expanded laterally from 7^th^ antennomere onwards (Fig. 15I); vertex and frons roughly punctate, postgenae rugose. ***Pronotum***: oblong, length-width ratio ca. 1.5, constricted posteriorly; surface finely and densely punctate, clothed with long, yellow hairs. ***Elytra***: oblong, sides subparallel, length/width ratio ca. 2.6, vested with grayish white setae; wedge-shaped protuberance present on inner surface; PAP arranged in ten rows, AAP absent; interspace between 2^nd^–3^rd^PAP rows ca. 2 × as wide as the punctation diameter; asetiferous punctations decreasing in size postmedially, and completely vanished at apical third (Fig. 10C). ***Legs***: outer apex of protibial apex slightly extending obliquely, not forming a distinct tooth. ***Abdomen***: intercoxal process of the first ventrite grooved longitudinally; metacoxal abdominal depressions weekly ridged in anterior margin, perpendicular carinae absent. ***Male genitalia***: pygidium subquadrate, posterior margin rounded (Fig. 15G); sixth ventrite arciform, 3 × wider than length, posterior margin rounded, central membranous region oval, extending from anterior margin to posterior margin (Fig. 15H); tegmen tubiform, length ratio of phallobasic apodeme to phallobase ca. 1: 2.1 (Fig. 15A–C); parameres expanded, unhooked (Fig. 15B, b); interphallic plate shorter than half length of phallus (Fig. 15E); phallus apex knot-like, rounded (Fig. 15D, E). ***Female reproductive organs***: pygidium slightly wider than long, posterior margin rounded (Fig. 16B); sixth ventrite trapezoidal, 3 × wider than long, central membranous region broad, apical accessory membranous region absent (Fig. 16C); bursa copulatrix clearly defined; spermathecal gland with a top tail of medium length (Fig. 16A, spg); spermathecal duct slender; spermathecal capsule slender, tapering to the tip, length/width ratio = 3.6 (Fig. 16A, sp).

##### Distribution.

Malaysia (Peninsular Malaysia).

##### Etymology.

Refer to the highly regular elytral asetiferous punctations of this species.

#### Gastrocentrum xiaodongi

Taxon classificationAnimaliaColeopteraCleridae

sp. nov.

BA64333F-A7F0-59F6-AA0A-746D1F3D6880

http://zoobank.org/B249CD1A-1BBC-4698-AA3C-961FF427B647

Figures 5 , 10D , 16D–H , 25

##### Holotype.

China: “Xizang (Tibet), Jilongxian [Gyirong county], 1785m, Xinjiangcun, 2019.VI.28, leg. X-D. YANG / Holotype: Gastrocentrum xiaodongi sp. nov. Yang & Yang, 2020” (CCCC, female, Fig. 5). **Paratypes**. **Nepal**: Manaslu Mts., E slope of Ngadi Khola valley, 2000–2300 m, 14–16.V.2005, leg. J. Schmidt, 28°22'N, 84°29'E (RGCM, 1 male); W-Nepal, Modi Khola, Bhakta B.; Banthanti – 2500 – Landrung – 1600 m, 2.VI.1984 (NHMB, 1 female).

##### Diagnosis.

This new species is different from *G. regulare* sp. nov. by: antennae expanded laterally from 8^th^ antennomere onwards; elytral asetiferous punctations stop by middle (Fig. 10D); elytral inner surface without wedge-shaped protuberance; intercoxal process of first abdominal ventrite not grooved (Fig. 20C, ip); spermathecal capsule thicker, rounded distally (Fig. 16F, sp). The new species also looks similar to *G. zayuense* sp. nov. and *G. gaoligongense* sp. nov. at first glance, but it differs from the latter two species by: elytral asetiferous punctations somewhat larger and reaching lateral margins (Fig. 10D), female antennae expanded laterally from 8^th^ antennomere onwards (Fig. 16H). It also differs from *G. gaoligongense* sp. nov. by elytral inner surface without a wedge-shaped protuberance.

##### Description.

***General appearance*:** length 14 mm, brown, a little slenderer than previous species. ***Head***: including eyes slightly broader than pronotum; eyes moderately large, distance between eyes slightly greater than the transverse diameter of eye; female antennae expanded laterally from 8^th^ antennomere onwards (Fig. 16H); vertex and frons rugose, densely punctate, postgenae rugose. ***Pronotum***: oblong, length/width ratio ca. 1.6, constricted posteriorly; surface finely and densely punctate, clothed with long, yellow hairs. ***Elytra***: oblong, sides subparallel in basal half and weekly widened in apical half, length/width ratio ca. 2.4, vested with yellow setae; wedge-shaped protuberance absent on inner surface; PAP only present on basal half in ten rows, AAP absent, PAP a little larger than those in *G. regulare*, *G. zayuense*, and *G. gaoligongense*; interspace between 2^nd^-3^rd^PAP rows greater than punctation diameter (Fig. 10D). ***Legs***: outer apex of protibia not extending outwards. ***Abdomen***: intercoxal process of the first ventrite not grooved longitudinally; metacoxal abdominal depressions weekly ridged in anterior margin, perpendicular carinae absent. ***Male genitalia***: not studied. ***Female reproductive organs***: pygidium slightly broader than long, posterior margin rounded (Fig. 16D); sixth ventrite semi-circle, central membranous region broad, apical accessory membranous region absent (Fig. 16E); both dorsal and ventral lamina have three incisions; bursa copulatrix clearly defined; spermathecal gland with a short top tail; spermathecal duct slightly inflated distally; spermathecal capsule rounded in apex, length/width ratio = 2.0. (Fig. 16F, G).

##### Distribution.

China (Xizang, Gyirong), Nepal.

##### Etymology.

We are pleased to dedicate this species to its collector and our friend, Mr Yang Xiaodong.

#### Gastrocentrum zayuense

Taxon classificationAnimaliaColeopteraCleridae

sp. nov.

D62CD9A2-59CD-587A-A24D-0DF0D9C11CC5

http://zoobank.org/BF8965BA-6A05-4D76-B545-2A844A5453FA

Figures 6 , 10F-H , 17 , 18 , 21 , 24 , 25

##### Holotype.

China: Xizang Autonomous Region, Nyingchi prefecture, Zayü County, Zhowagoin, 2011.VII.1, BI Wenxuan leg, 2500 m / Holotype: Gastrocentrum zayuense sp. nov. Yang & Yang, 2020 (CBWX, male, Fig. 6); **Paratypes.** same as holotype (CBWX, 15 ex.); same as holotype but collected by LIU Ye on 2011.VII.3 (IZAS, 9 ex.); same but collected by Yang Xiaodong on 2011.VII.3 (CCCC, 1 ex.).

##### Diagnosis.

This species differs from *G. gaoligongense* sp. nov. by elytral inner surface without wedge-shaped protuberance; tegmen apices with ventral surface streamlined in lateral view (Fig. 17a), female bursa copulatrix clearly defined, much narrower than vagina, distal part of spermathecal capsule short, length/width ratio < 2.5 (Fig. 18A).

##### Description.

***General appearance*:** length 13–18 mm, somewhat slenderer than *G. regulare*, light brown. ***Head***: including eyes feebly broader than pronotum; eyes moderately large, distance between eyes slightly greater than the transverse diameter of eye; gular suture slightly convergent in anterior; female antennae broadly expanded laterally from 7^th^ antennomere onwards, while male 7^th^ antennomere less expanded (Fig. 17H); vertex and frons finely punctate, postgenae rugose. ***Pronotum***: oblong, length/width ratio ca. 1.5, constricted posteriorly; surface finely and densely punctate, clothed with long, yellow hairs. ***Elytra***: oblong, sides subparallel in basal half and a little widened in apical half, length/width ratio ca. 2.5, vested with light yellow setae; wedge-shaped protuberance absent on inner surface; elytron smooth without asetiferous punctations or with very few asetiferous punctations, number of PAP ranged from 0~27 (n = 26), present on elytral basal disc in six rows in maximum, AAP absent (Fig. 10F–H). ***Legs***: outer apex of protibia not extending outwards. ***Abdomen***: intercoxal process of the first ventrite not grooved longitudinally (Fig. 20C); metacoxal abdominal depressions weekly ridged in anterior margin, perpendicular carinae absent. ***Male genitalia***: pygidium with posterior margin rounded (Fig. 17F); sixth ventrite arciform, width twice length, posterior margin rounded, central membranous region small, rhombic, extending from anterior margin to half-length of the ventrite (Fig. 17G); tegmen tubiform, length ratio of phallobasic apodeme to phallobase ca. 1: 3.2 (Fig. 17A, B); parameres hooked, ventral surface of the hook streamlined in lateral view (Fig. 17a); interphallic plate shorter than half length of phallus (Fig. 17D); phallus apex knot-like, approximately as long as wide (Fig. 17C, D). ***Female reproductive organs***: pygidium subquadrate, posterior margin rounded (Fig. 18B); sixth ventrite trapezoidal, twice as broad as long, rounded posteriorly, central membranous region broad and extending posteriorly at sides, apical accessory membranous region absent (Fig. 18C); bursa copulatrix clearly defined; spermathecal gland with a top tail of medium length; spermathecal duct inflated distally where continuous with spermathecal capsule; spermathecal capsule simple or feebly bifurcate (Fig. 18D–J), distal part of spermathecal capsule short, length/width ratio < 2.5 (Fig. 18A, E–J).

##### Variation.

All examined specimens are from exactly same locality, they vary individually in the number of punctations on one elytron from zero to 27, and spermatheca apex being simple or feebly bifurcate distally.

##### Distribution.

China (Xizang, Zayü).

##### Ecology.

Habitat is shown in Fig. 24. The specimens were collected on the tree trunk at night.

##### Etymology.

The new species is named after its type locality.

#### Gastrocentrum gaoligongense

Taxon classificationAnimaliaColeopteraCleridae

sp. nov.

D6BC2143-ECBE-50DF-83F0-3866C60FDA20

http://zoobank.org/A1EC3952-6CAA-4F94-9222-3D5324686AA2

Figures 7 , 10E , 19 , 22E , 25

##### Holotype.

China: “CHINA, Yunnan Province, Fugong Co., Lishadi town, Shibali village, roadside, 27.16520°N, 98.77980°E / 2530 m, 2004.5.5, night, Liang H-B, Li X-Y, coll., California Academy &IOZ., Chinese Acad Sci / IOZ(E) 1890507 / Holotype / Gastrocentrum gaoligongense sp. nov. Det. Yang G.Y. 2020” (IZAS, male) (Fig. 7). **Paratypes**. CHINA: Yunnan, Gongshan, Menggaguo, 2800 m, Light, 2016.VII.8, Yu-Tang Wang leg. (CCCC, 1 male, 1 female); same but 2016.VI.30 (CCCC, 1 male); same but 2016.VI.24 (CCCC, 1 female).

##### Diagnosis.

The new species differs from *G. zayuense* sp. nov. by: elytra with a pair of wedge-shaped protuberance on inner surface; tegmen apices bulged on ventral surface (Fig. 19b); female bursa copulatrix not differentiated, merely a swollen continuation of the vagina, distal part of spermathecal capsule, long and slender, length/width ratio > 5 (Fig. 22E).

##### Description.

***General appearance*:** length 13–19 mm, slenderer than all the other species, dark brown. ***Head***: including eyes slightly broader than pronotum; eyes moderately large, distance between eyes slightly longer than the transverse diameter of eye; gular suture slightly convergent in anterior; female antennae broadly expanded laterally from 7^th^ antennomere onwards, while male 7^th^ antennomere less expanded (Fig. 19H); vertex and frons with dense and somewhat coarse punctations, postgenae rugose. ***Pronotum***: oblong, length/width ratio ca. 1.6, constricted posteriorly, faintly constricted anteriorly; surface finely and densely punctate, clothed with long, yellow hairs. ***Elytra***: oblong, sides subparallel in basal half and weekly widened in apical half, length/width ratio ca. 2.6, vested with light yellow setae; wedge-shaped protuberance present on inner surface; elytra with very few asetiferous punctations, number of PAP on each elytron ranged from 2~39 (n = 5), present on elytral basal disc in five rows in maximum, AAP absent (Fig. 10E). ***Legs***: outer apex of protibia not extending outwards. ***Abdomen***: intercoxal process of the first ventrite not grooved longitudinally; metacoxal abdominal depressions weekly ridged in anterior margin. ***Male genitalia***: pygidium with posterior margin rounded (Fig. 19F); sixth ventrite arciform, width twice length, posterior margin rounded, central membranous region small, extending from the anterior margin, not reaching half-length of ventrite (Fig. 19G); tegmen tubiform, length ratio of phallobasic apodeme to phallobase ca. 1: 3.2 (Fig. 19A–C); parameres hooked, ventral surface of the hook bulged in lateral view (Fig. 19b); interphallic plate shorter than half length of phallus (Fig. 19D); phallus apex knot-like, slightly longer than wide (Fig. 19D, d). **Female reproductive organs**: pygidium slightly broader than long, posterior margin rounded; sixth ventrite widely trapezoidal, 2.5 × broader than long, rounded posteriorly, central membranous region broad, apical accessory membranous region absent; bursa copulatrix unclearly defined, merely swollen continuation of vagina; spermathecal gland with a top tail of medium length; spermathecal duct long and slender; spermathecal capsule feebly bifurcate in sub-apex; distal part of spermathecal capsule long and slender, length/width ratio > 5 (Fig. 22E).

##### Distribution.

China (Yunnan).

##### Etymology.

The holotype and paratypes of this new species were collected from two sites of the Gaoligong Mountains in Yunnan Province, China. The specific name is an adjective that refers to this mountain.

#### Gastrocentrum brevicolle

Taxon classificationAnimaliaColeopteraCleridae

(Pic, 1940)

50B6A038-3BA2-5C46-B5CE-7657C5EB697A

Exocentrum brevicolle Pic, 1940: 3 (type locality: “Ceylan”); Corporaal, 1950: 55 (Gastrocentrum); Mawdsley, 1999: 271 (Sri Lanka); Gerstmeier, 2005: 56.

##### Note.

This species was not studied because specimens were unavailable. Gerstmeier (2005) stated that the position of this species in the genus *Gastrocentrum* is doubtful because its pronotum was not elongated, but rather spherical.

#### Tillus nitidus

Taxon classificationAnimaliaColeopteraCleridae

(Schenkling, 1916) comb. nov.

0A35AF55-3846-5593-A512-65A909FFD6F9

Figure 8

Gastrocentrum nitidum Schenkling, 1916: 117 (type locality: “Banshoryo-Distrikt, Sokutsu”, Taiwan); Corporaal, 1950: 55 (catalogue).

##### Type specimen examined. Holotype.

“Banshoryo Distr. Sokutsu (Formosa), H. Sauter VII. 1912 / Holotypus / Schenkling det. / Gastrocentrum nitidum Schklg. Typus!” (SDEI, female; Fig. 8).

##### Notes.

This species is transferred to the genus *Tillus* for its claw with two inner denticles (basal denticle trigonal). This type of claw was imaged in Burke (2017: 179, fig. B) of the species *Cymatodera balteata*.

#### Isocymatodera atricolor

Taxon classificationAnimaliaColeopteraCleridae

(Pic, 1935)

CF02B547-C609-5A97-871E-6BA49AB50156

Figures 9 , 20F , 22A , 22B

Strotocera atricolor Pic, 1935: 6 (type locality: “Indochine”); Gerstmeier, 2009: 5 (Isocymatodera).

##### Type specimens examined. Syntypes.

“[...] / voi Tillus / Type [printed] Strotocera atricolor nouv. [hw. by Pic] / ?abyssinie / voi Strotocera / type [hw. by Pic] / Paris” (MNHN, 1 female; Fig. 9); “Baria / Baria (Cochinchina) / acq. 1930 coll. Ch. Madon (Le Moult) / Cotype: Strotocera atricolor Pic, 1934 / Strotocera atricolor Pic, 1935, ZMAN type 1939.1” (ZMAN, 1 female).

##### Other specimens.

**China.** Hainan, Changjiang County, Bawangling Forest Nature Reserve (IZAS, 6 ex.); Ledong County, Jianfengling Tropical Rainforest National Park (IZAS, 4 ex.); Baisha County (IZAS, 1 ex.). **Vietnam**. “Baria [...]/Baria (Cochinchina) / acq. 1930 coll. Ch. Madon (Le Moult) / Homotype: (Corporaal comp.): Strotocera atricolor Pic” (ZMAN, 1 ex.).

##### Note.

This species is recorded from China for the first time, and hence we provide a short note here.

### Phylogenetic relationships

**Figure 23.**
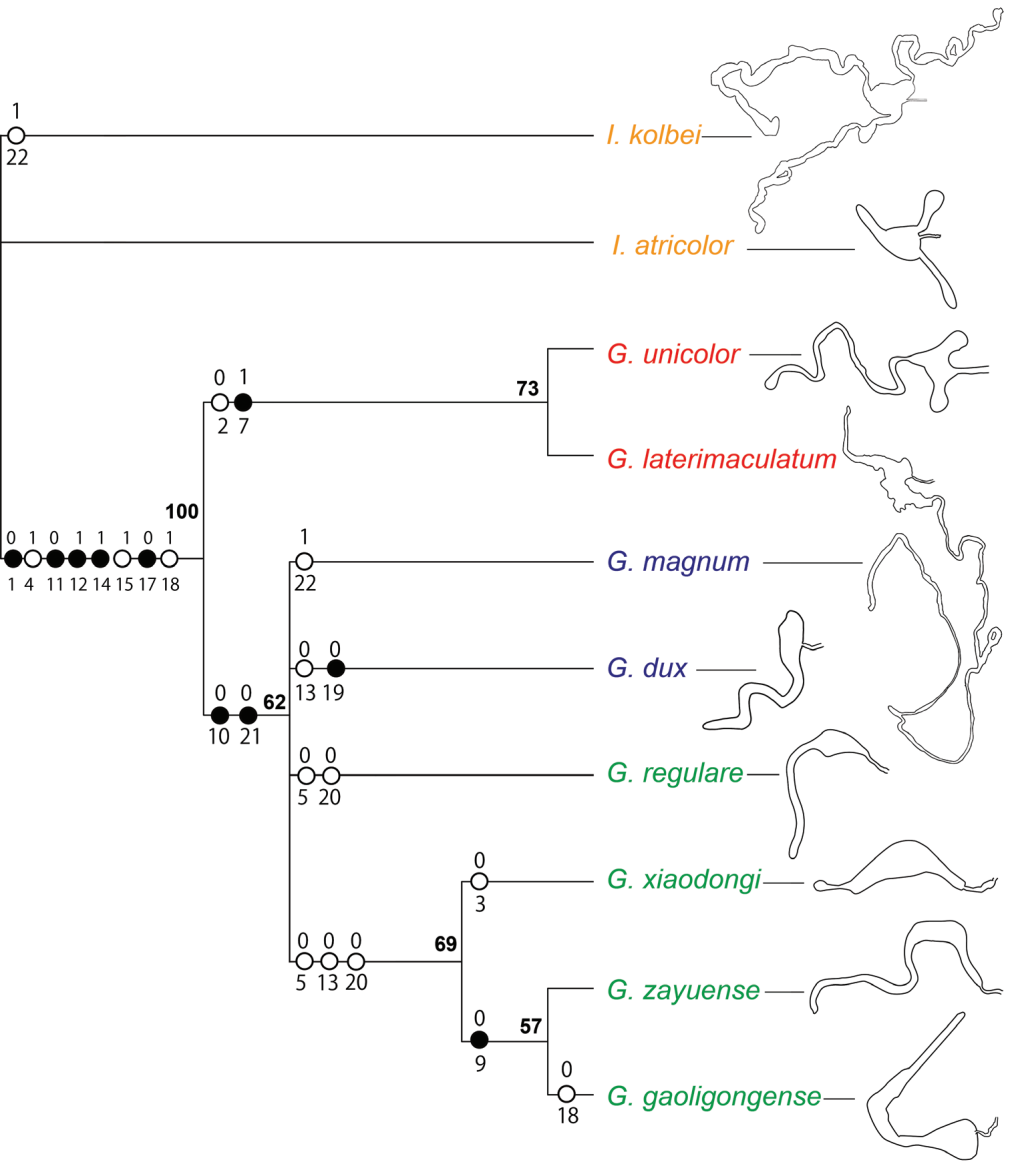
A preliminary phylogenetic analysis of *Gastrocentrum*, showing 50% majority-rule consensus MP tree. Only unambiguous characters are shown. Black circles represent characters having a CI of 1.000 while each state is derived only once, whereas white circles represent characters having a CI less than 1.000 while each state is derived more than once. Bootstrap support values are given at nodes. Female spermathecal glands are illustrated with top tail orientated to left side, but it is not known in *G. laterimaculatum*.

A phylogenetic analysis resulted in eight most parsimonious trees in PAUP* (L = 36, CI = 0.611, RI = 0.659, RC = 0.402) (Fig. 23). The eight species comprise two clades, the first clade including *G. unicolor* and *G. laterimaculatum* (bootstrap value 73), and the second clade including the other six species (bootstrap value 62). The monophyly of the first clade is supported by the 7^th^ antennomere not extended laterally (character 2: 0; CI = 0.500) and AAP distinctly arranging in two rows (character 7: 1; CI = 1.000). The second clade is supported by inclusive synapomorphies: protibial outer-apical tooth absent (character 10: 0; CI = 1.000) and female spermathecal gland not having two lateral tails (character 21: 0; CI = 1.000).

The second clade forms a polytomy consisting of *G. magnum* sp. nov., *G. dux*, *G. regulare* sp. nov., and a moderately supported sub-clade representing the remaining ingroup species. The monophyly of this sub-clade (bootstrap value 69) is supported by elytral interspace between 1^st^-2^nd^PAP rows without AAP (character 5: 0; CI = 0.500), intercoxal process of first abdominal ventrite not grooved (character 13: 0; CI = 0.500) and female spermathecal gland without any lateral tail (character 20: 0; CI=0.500). Within this sub-clade, *G. xiaodongi* sp. nov. is the sister group of *G. zayuense* sp. nov. + *G. gaoligongense* sp. nov.; the monophyly of the latter is supported by elytral punctations not reaching lateral margins (character 9: 0; CI = 1.000).

### Significance of female reproductive organs

Female reproductive organs are inferred to have taxonomic and phylogenetic importance in genus *Gastrocentrum*.

In certain Oriental genera of Tillinae, the vagina is equipped with a pair of sclerites or a joint sclerite, such as in *Tillus* (Kolibáč 1989: 17, figs 46, 47), *Isocymatodera* (Fig. 22A, C, D) and *Cladiscus* (unpublished data), but this sclerite is absent in all the species of *Gastrocentrum*.

In the present study, we find that almost all the species in *Gastrocentrum* have a clearly defined bursa copulatrix (Figs 12, 14, 16, 18), the only exception being *G. gaoligongense* (Fig. 22E). This disproves the idea of Opitz (2010: 51) that the presence or absence of a bursa copulatrix is consistent within stable genera.

The morphology of the spermathecal gland was rarely extensively studied previously in Cleridae. In *Gastrocentrum*, we find that this structure was phylogenetically significant at the infra-generic level (Fig. 23). The three-tailed spermathecal gland occurring in *G. unicolor* and the outgroup *Isocymatodera* was supposed to be plesiomorphic in *Gastrocentrum* (Fig. 23). In *G. magnum* and *G. dux* one lateral tail was lost, while in *G. regulare*, *G. zayuense*, *G. xiaodongi*, and *G. gaoligongense*, two lateral tails were lost. The absence of the top tail was autapomorphic for *G. dux*.

**Figure 24.**
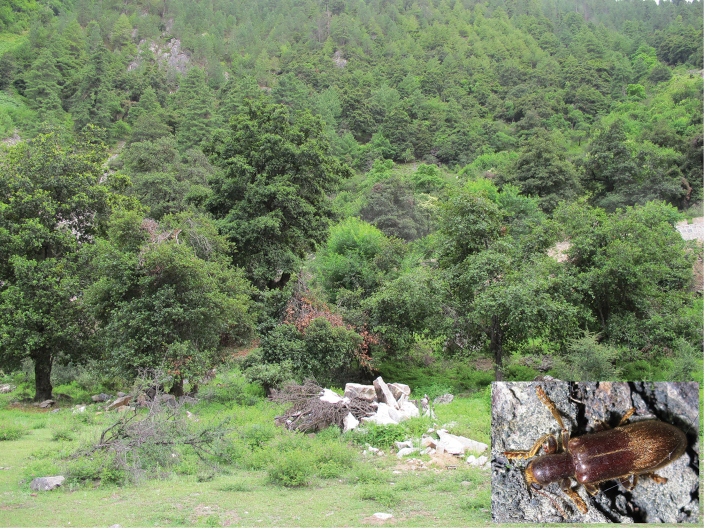
Habitat of *G. zayuense* sp. nov. Photograph by BI Wenxuan.

The shape of the spermathecal capsule was believed by Opitz (2010: 51) to be consistent within stable genera, however, we found that this structure was different among several *Gastrocentrum* species and thus was of some value for species-level identification, for example, the spermathecal capsules were tapered in *G. regulare* (Fig. 16A), barrel-shaped in *G. xiaodongi* (Fig. 16G), short in *G. zayuense* (Fig. 18A), and long and slender in *G. gaoligongense* (Fig. 22E).

**Figure 25.**
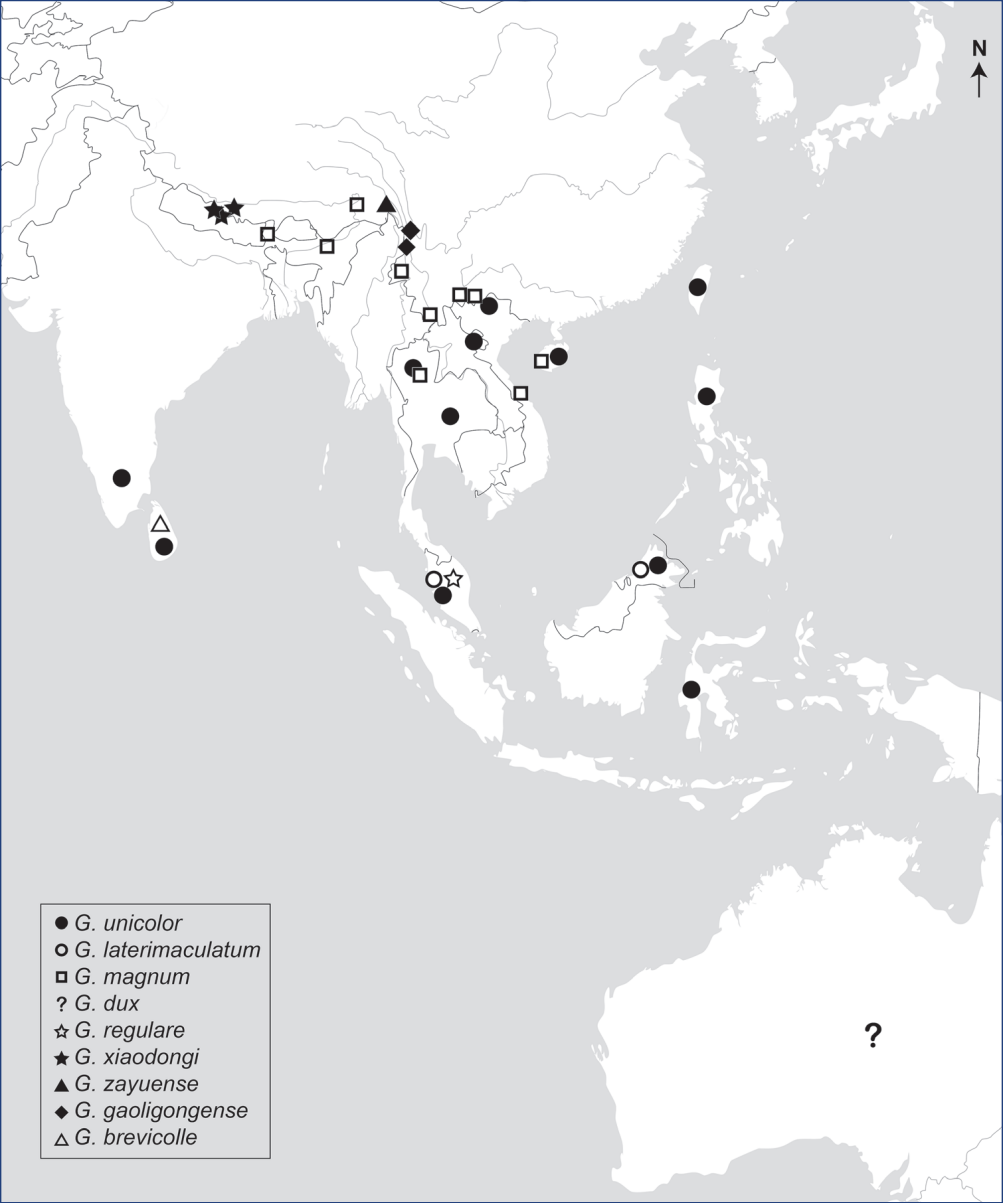
Geographical distribution map of the genus *Gastrocentrum*.

## Supplementary Material

XML Treatment for Gastrocentrum

XML Treatment for Gastrocentrum unicolor

XML Treatment for Gastrocentrum laterimaculatum

XML Treatment for Gastrocentrum magnum

XML Treatment for Gastrocentrum dux

XML Treatment for Gastrocentrum regulare

XML Treatment for Gastrocentrum xiaodongi

XML Treatment for Gastrocentrum zayuense

XML Treatment for Gastrocentrum gaoligongense

XML Treatment for Gastrocentrum brevicolle

XML Treatment for Tillus nitidus

XML Treatment for Isocymatodera atricolor
